# Psychological characteristics and functional abilities in older adults with subjective cognitive complaints: A potential new target for early intervention

**DOI:** 10.1016/j.inpsyc.2025.100066

**Published:** 2025-03-24

**Authors:** Jaclyn M. Fox, Danielle J. Harvey, Christine N. Pons, Yichu Chen, Samina Rahman, Michael J. Ryan, Jagnoor Randhawa, Alyssa M. Weakley, Michelle L. Chan, Maureen Schmitter-Edgecombe, Sarah Tomaszewski Farias

**Affiliations:** a University of California, Davis Department of Neurology, Sacramento, USA; b University of California, Davis Department of Public Health Sciences, Davis, USA; c University of California, Davis School of Medicine, Sacramento, USA; d Washington State University, Department of Psychology, Pullman, USA

**Keywords:** Aging, Subjective cognitive decline, Positive psychological characteristics, Positive and negative affect, Functional abilities

## Abstract

**Objectives:**

Positive psychological characteristics may help to bolster daily functioning in older adults even in the presence of subjective or subtle objective cognitive weaknesses. The purpose of the present study was to examine which specific psychological characteristics may contribute to better functional abilities, independent of cognitive performance, among a sample of older adults with subjective cognitive decline (SCD).

**Design, Setting, and Participants:**

Participants included 277 older adults recruited from the community who were enrolled in a randomized controlled trial examining the impact of training in memory support strategies and healthy lifestyle behaviors. The current study was based on baseline data prior to intervention randomization.

**Measurements:**

Linear regression was used to assess associations between positive psychological characteristics (positive affect, life satisfaction, purpose in life, resiliency, self-management of health, and perceived competence), one negative psychological characteristic (negative affect), and functional abilities controlling for demographic variables. A final model assessed for associations between functional abilities and any of the significant characteristics identified in the linear regressions while controlling for global cognition (measured with the Modified Neuropsychological Test Battery composite score), depression, and demographic variables.

**Results:**

In the individual regressions, most of the psychological characteristics were significantly associated with functional abilities. In the final model, positive and negative affect remained significantly associated with functional abilities.

**Conclusions:**

This is one of the first studies to show that greater positive affect is associated with better functional abilities, even after controlling for objective cognitive performance. Positive affect in older adults may represent a modifiable target for intervention.

## Introduction

Cognitive impairment in older adults is a significant public health concern associated with a host of negative outcomes including loss of independence and increased care needs [1,2]. While cognitive impairment is a major cause of declines in instrumental activities of daily living in older adults, noncognitive factors can also impact the ability to function independently, and many of these factors are modifiable, making them amenable to intervention strategies [3]. Depression is one of the most studied noncognitive factors, and depression in older adults is associated with ‘excess disability’ beyond what would be expected solely due to concomitant health conditions [4–6]. In contrast, positive psychological characteristics (PPCs) may enhance resilience to functional decline and buffer some of the effects of incipient cognitive decline (i.e., functional reserve [7,8]). Limited work has examined PPCs associated with functional outcomes among older adults. One previous study among individuals with human immunodeficiency virus (HIV) demonstrated that self-rated resiliency was associated with greater independence in instrumental activities of daily living (IADLs) and fully mediated the relationship between cognition and function [9].

A number of PPCs are associated with enhanced health outcomes, including reduced disease risk. Perhaps most studied in this regard are the Big Five personality traits. Specifically, conscientiousness (the propensity to be organized, hard-working, and goal-directed), and to a lesser extent openness to experience and extraversion, have been associated with a variety of health outcomes [10–13], including dementia risk [14]. However, personality traits are relatively broad and stable and may be less amenable to modification. There have been a host of other PPCs that have also been associated with health outcomes. The propensity to experience positive affect has been shown to positively impact many health outcomes and studies have shown higher levels of positive affect is often associated with reduced risk of negative health outcomes and conditions (e.g., mortality, cardiovascular disease, diabetes, HIV, and cancer) (see Pressman et al. [15] for a review). While studies specific to older adults remain limited, work by our group [16] and others [17–19] has shown that positive affect is associated with better cognitive outcomes among older adults. Although, the impact of positive affect on daily functioning has not been well studied, one study showed that among older adults with arthritis, high positive affect was associated with reduced risk of functional decline over a two-year period [20].

Other PPCs (e.g., sense of life satisfaction, resiliency, life purpose, self-management of health, and perceived competence) have been examined in association with health outcomes. For example, studies have found that life purpose is positively associated with cognitive performances in older adults [21,22] and negatively associated with risk of memory impairment up to nine years later [23]. Among individuals with Alzheimer’s disease, those with high levels of life purpose exhibited better cognitive functioning than those with low levels regardless of the number of neurofibrillary tangles found in their brain post-mortem [24]. Limited work has also found positive associations between cognitive outcomes and perceived competence [22,25], life satisfaction [26], and self-rated resilience [27]. Finally, higher levels of self-management of health have been linked to a slower rate of cognitive decline [28,29], perhaps because these individuals may engage in better health related behaviors. Furthermore, a decline in self-management of health has been associated with cognitive decline in older adults [30].

In sum, there is accumulating evidence that certain psychological characteristics may have a positive impact on cognitive aging. However, the impact of modifiable PPCs on *daily functioning* among older adults is largely unknown. This represents a critical knowledge gap, given the importance of functional independence to most older adults [31], its association with other poor outcomes such as nursing home placement and higher healthcare costs [32,33], and the potential for identifying new treatment targets to support independence. Within this context, the purpose of the current study was to examine specific psychological characteristics (e.g., positive affect, life satisfaction, purpose in life, resiliency, self-management of health, and perceived competence) that may contribute to *better* functional abilities regardless of current cognitive ability. In addition, we examined one possible modifiable psychological characteristic that may contribute towards *worse* functional abilities (negative affect). The current study examines psychological characteristics in older adults who report subjective cognitive decline (SCD), a known risk factor for the development of cognitive impairment and dementia [34,35] that can also be associated with subtle difficulties in daily functioning [36]. We hypothesized that a variety of PPCs would be associated with better functional abilities, independent of cognition.

## Methods

### Participants

The current study sample included 277 community-dwelling English-speaking participants age 65 + with SCD that were enrolled in a National Institute of Health (NIH)-funded randomized controlled trial (RCT) teaching memory support strategies and healthy lifestyle behaviors (see Tomaszewski Farias et al. [37] for further methodological details). SCD was defined by endorsement of “a change in your memory or other aspect of thinking in the last one to three years” with normal performance on a cognitive screening measure (the Telephone Interview for Cognitive Status [TICS] [38]) and the ability to live and function independently in the community. To be enrolled in the study, participants also had to have a relatively low level of engagement in healthy lifestyle behaviors (defined as not engaging in exercise resulting in sweating or elevated heart rate ≥3 times per week). Exclusion criteria included a known diagnosis of cognitive impairment or dementia along with other major neurological condition (such as Parkinson’s disease) or major psychiatric disorders such as schizophrenia or active severe depression (scoring greater than 15 on the Patient Health Questionnaire [PHQ-9] [39]) or suicidal ideation. The institutional review board at the University of California, Davis approved the study procedures, and all participants provided written informed consent prior to participation. This study utilized baseline data from the trial, before the start of the intervention.

### Measurement of psychological characteristics

Psychological characteristics were measured through a variety of self-report questionnaires. Positive and negative affect were measured by the Positive and Negative Affect Scale (PANAS) [40]. The PANAS consists of 10 items measuring positive affect (i.e., feeling interested, excited, strong, enthusiastic, proud, alert, inspired, determined, attentive, and active) and 10 items measuring negative affect (i.e., feeling distressed, upset, guilty, scared, hostile, irritable, ashamed, nervous, jittery, and afraid). Participants rate how often they typically experience these items on a scale from 1 (very slightly or not at all) to 5 (extremely). Satisfaction with life was measured by the Satisfaction With Life Scale (SWLS) [41], which consists of five items related to life satisfaction (e.g., In most ways my life is close to my ideal, The conditions of my life are excellent, etc.). Participants rate how much they agree with each item on a scale from 1 (strongly disagree) to 7 (strongly agree), with higher scores representing higher life satisfaction. Resiliency was measured by the Brief Resiliency Scale [42], which consists of six items related to how the participant responds to negative events (e.g., I tend to bounce back quickly after hard times, It does not take me long to recover from a stressful event, etc.). Participants rate agreement with each item on a scale from 1 (strongly disagree) to 5 (strongly agree), with higher scores representing more resiliency. Life purpose was measured by the Purpose in Life dimension of the Psychological Well-Being Scale [43], which consists of four items related to life purpose (e.g., I feel good when I think of what I’ve done in the past and what I hope to do in the future, I have a sense of direction and purpose in life, etc.). Participants rate how much they agree with each item on a scale from 1 (strongly disagree) to 5 (strongly agree), with higher scores representing more purpose in life. Self-management of health was measured by the Patient Activation Measure (PAM-13) [44], which consists of thirteen items regarding their self-perceived ability and knowledge in managing health conditions (e.g., I am confident that I can follow through on medical treatments I need to do at home, I understand the nature and causes of my health condition(s), I know the different medical treatment options available for my health conditions, etc.). Participants rate how much they agree with each item on a scale from 1 (strongly disagree) to 4 (strongly agree), with higher scores representing more self-management of health. Perceived competence was measured by an adapted version of the Perceived Social Competence Scale (social changed to healthy brain aging) [45], which consists of four items measuring a participant’s belief in their ability to maintain brain health behaviors (e.g., I feel confident in my ability to maintain healthy brain aging behaviors on a regular basis, I am able to maintain healthy brain aging behaviors long term, etc.). Participants rate how true each statement is on a scale from 1 (not at all true) to 7 (very true), with higher scores representing higher perceived competence.

### Cognition

Cognition was evaluated with the POINTER-modified Neuropsychological Test Battery (PmNTB) [46] derived from the Neuropsychological Test Battery [47]. The PmNTB has been used in multiple studies and is designed to generate a global cognitive composite score using outcomes from several cognitive domains that have been shown to respond to lifestyle interventions [48]. Specifically, the global cognitive score is comprised of equally weighted scores from Processing Speed, Executive Function, and Episodic Memory composites. The Processing Speed composite includes Trail Making Test Part A and Digit Symbol Substitution. The Executive Function composite includes Number Span on Digits Backward and Sequencing, Semantic and Phonemic Verbal Fluency, and Trail Making Test Part B. The Episodic Memory composite includes The Free and Cued Selective Reminding Test and Story Memory. Each composite was calculated by averaging standardized scores (z-scores). Z-scores were calculated with cohort-wide mean and standard deviation scores at baseline and were adjusted such that positive scores always reflect better cognitive function. The global cognition score was then calculated by averaging the three composite scores.

### Depression

Depression was measured through the Center for Epidemiologic Studies Depression Scale (CES-D) [49]. The CES-D is a self-report measure comprised of 20 depressive symptoms. Participants rate the frequency of these symptoms over the past week on a scale from 1 (rarely or none of the time/less than 1 day) to 4 (most or all of the time/5–7 days), with higher scores representing greater levels of depression.

### Outcome: Functional Abilities (IADLs)

Functional abilities were obtained through the Activities of Daily Living-Prevention Instrument (ADL-PI) [50] self-report form. The ADL-PI consists of 15 questions about daily functional abilities. Abilities assessed include financial management (e.g., paying bills, withdrawing or obtaining money, etc.), driving/taking public transportation, using appliances or making household repairs, doing laundry, shopping, preparing meals or snacks, finding personal belongings around the house, writing letters or lists for others, following television shows or movies, remembering current events, making phone calls, taking medications, planning and organizing complex activities (such as travel, errands, etc.), and completing complex hobbies (such as reading, gardening, etc.). Participants rate how well they are managing these everyday tasks in the past three months with the following options: As well as usual with no difficulty, With a little difficulty, With a lot of difficulty, or You did not do this activity. Lower scores represent better functional abilities.

### Statistical analyses

Linear regression was used to assess associations between the six self-report psychological characteristics (positive affect, negative affect, satisfaction with life, life purpose, self-management of health, and perceived competence) and the outcome variable (functional abilities). The functional abilities measure was log transformed prior to analyses (after shifting by a small number to account for zeros) to meet the assumptions of the models. All models included age, gender, and education. Model building began with simple models that included one key independent variable (i.e., psychological characteristic) in addition to the demographic variables. A final model was then built that included all independent variables that met a statistically significant level of association in the simple models (i.e., set at p < .05). There were two control variables of interest, global cognition and depression, that would be included in the final model if they were found to be individually associated with functional abilities. Correlations between all independent variables were checked to assess for collinearity with correlations found between depression and multiple psychological characteristics, including positive affect, negative affect, life satisfaction, life purpose, and resiliency (see Table 1). Model diagnostics were used to assess underlying assumptions with no additional findings of concern. Ten participants were excluded from the final models due to missing data. All analyses were conducted in R and a p-value less than 0.05 was considered statistically significant.

## Results

Table 2 includes the demographics, psychological characteristics, and functional ability of the sample at study baseline. Overall, the group was well educated (mean 15.9 years) and over 50 % were female. Over a quarter of the sample was comprised of individuals from traditionally under-represented groups.

We first examined the association between each psychological characteristic individually and the functional outcome, while controlling for demographics. As presented in Table 3, positive affect, life satisfaction, resiliency, life purpose, and self-management of health were all identified in the initial models as having a positive association with functional abilities and negative affect was identified in the initial model as having a negative association with functional abilities while controlling for demographic variables. Perceived competence was the only psychological characteristic not significantly independently associated with functional abilities. Additionally, as anticipated, both depression (negatively associated) and global cognitive function (positively associated) were also significantly associated with functional abilities.

We then examined a multivariate model to test for potential independent associations between each psychological characteristic that was found to be significantly associated with functional abilities in the individual models, while controlling for global cognition, depression, and demographics. As presented in Table 4, positive affect and negative affect both remained significantly associated with functional abilities. Specifically, higher positive affect was associated with better functional abilities and higher negative affect was associated with worse functional abilities. Global cognition no longer reached the threshold of statistical significance in its association with functional abilities. None of the other psychological characteristics or depression remained significantly associated with functional abilities.

Due to correlations between depression and multiple psychological characteristics, a follow-up model was run without depression to determine if collinearity was a concern. As presented in Table 4b, removing depression from the model did not change the findings as positive affect and negative affect remained significantly associated with functional abilities. Global cognition no longer reached the threshold of statistical significance in its association with functional abilities.

## Discussion

The current study revealed that multiple PPCs are individually associated with functional abilities. In the joint model, affective ratings remained associated with functional abilities, specifically positive affect was associated with better functioning and negative affect with worse functioning, even when controlling for current cognitive ability and depression. These findings suggest that certain psychological characteristics, both positive and negative, may independently relate to daily functional abilities in older adults.

In the individual regression models, greater degrees of various PPCs, including life satisfaction, purpose in life, resiliency, and self-management of health, were significantly associated with better functional abilities. This finding is consistent with other research that higher levels of life satisfaction, purpose in life, resiliency, and self-management of health are also associated with better cognitive outcomes in older adults [24,26,28,29]. Higher levels of these characteristics may help older adults remain active, increase motivation, and better manage their physical and medical health, ultimately leading to preserved cognitive and daily functioning.

Given that positive affect was the only PPC that remained independently associated with functional abilities in the final joint model, it may be an important and common link across the PPCs on its association with better functioning and greater resiliency in aging. This finding aligns with prior research that found greater positive affect is associated with a host of more positive health outcomes and may be protective against negative health outcomes (e.g., mortality, cardiovascular disease, diabetes, HIV) [15]. Additionally, in a different cohort of older adults, we found that positive affect was also associated with better longitudinal cognitive outcomes, reducing the rate of cognitive decline over time [16]. The current study expands on this finding by indicating that positive affect is also associated with better functioning in a sample of older adults with SCD.

Several possible mechanisms may explain why higher levels of positive affect may be associated with better functional abilities. Positive affect does not simply involve feelings of happiness but also involves characteristics around interest/curiosity and feeling energized and motivated. These characteristics may be particularly important for staying engaged in activities as one ages. For example, one study found that encouraging feelings of hope and self-efficacy ameliorated the negative impact of depression on functioning in older adults [51]. Additionally, individuals with high positive affect may be more likely to persevere when challenges arise in daily functioning and may be more open to developing and using compensatory strategies. Finally, research suggests that individuals with high positive affect may have healthier lifestyles, which could, in turn, help promote functional abilities as one ages [20]. In this way, positive affect may contribute towards an individual’s level of functional reserve through multiple pathways.

Conversely, higher levels of negative affect were associated with worse functional abilities, even when a measure of depression was included in the model. Many previous studies have found that depression is associated with functional abilities [4–6]. Although depression and negative affect have overlapping core features, negative affect has been conceptualized as a separate construct. Negative affect is conceptualized as a state of negative emotions and includes aspects such as anger and anxiety, which may or may not be present in individuals with depression [40,52]. Our findings are in line with another previous study that found, even in the absence of significant depressive symptoms, greater negative affect has been associated with a higher risk of mild cognitive impairment or dementia in older women [53]. Negative affect has also been associated with worse cognition in older adults [54–56].

The current findings have important implications for the further development and evaluation of intervention strategies to promote greater functional independence in older adults by increasing positive affect and decreasing negative affect. To combat the impact of negative affect, it may be important to address not only mood symptoms but other factors, such as loneliness, social isolation, and anger, which may contribute to maintaining the negative affect [55–57]. To promote positive affect, it may be important to build up an individual’s self-efficacy (by providing realistic goals they can work towards) and encourage curiosity and learning. Additionally, multiple stress reduction and well-being interventions, including mindfulness, practicing gratitude, savoring positive events, performing small acts of kindness, and thinking optimistically, have been shown to improve positive affect in the general population, individuals with mental health diagnoses, and those with various medical conditions [15,57]. Mindfulness-based therapy has also been shown to increase other PPCs including life satisfaction and purpose in life in adults in the general population [58,59] and resiliency in adults in high stress work environments [60]. The impact of these stress reduction and well-being interventions are less studied in older adults, but the results of the current study suggest that these types of interventions could be effective in promoting prolonged functional independence among older adults with SCD.

### Limitations and future directions

The current study has several limitations. First, the current study is based on cross-sectional data so conclusions around causality cannot be made. It will be important to employ longitudinal follow up to examine whether these same (or other) psychological characteristics influence rates of decline in IADLs and whether they are protective against loss of independence. Additionally, as part of the inclusion criteria for the clinical trial, individuals had to be living and functioning independently in the community, so none of the participants had a frank loss of functional independence. This, however, is common in very early, preclinical samples and, regardless, we did find predictors of functional abilities. Future studies examining the impact of PPCs on functional abilities among other populations of older adults, including those with more obvious cognitive and functional impairment. Finally, the current study measured the psychological characteristics and functional abilities through self-report measures, which may not be accurate if participants lack insight into their own functional abilities or psychological characteristics. Therefore, future studies with both self- and informant-report of psychological characteristics and functional abilities may be helpful, particularly when examining older adult populations with cognitive impairment.

## Conclusion

In conclusion, we found that among a diverse group of older adults with SCD, higher levels of positive affect and lower levels of negative affect were associated with better functional abilities, even when controlling for current cognitive ability and depression. If confirmed in future work, particularly longitudinal analyses, there are important implications for intervention development. Specifically, interventions aimed at modifying affect may be effective in promoting prolonged functional independence among older adults with SCD.

## Figures and Tables

**Table 1:** Correlations Between Independent Variables.

	Positive Affect	Negative Affect	Life Satisfaction	Resiliency	Purpose in Life	Self-Management of Health	Perceived Competence	Depression	Global Cognition

Positive Affect	1.00								
Negative Affect	**−0.25**	1.00							
Life Satisfaction	**0.49**	**−0.35**	1.00						
Resiliency	**0.39**	**−0.51**	**0.43**	1.00					
Purpose in Life	**0.49**	**−0.31**	**0.50**	**0.44**	1.00				
Health Literacy	**0.24**	**−0.22**	**0.26**	**0.25**	**0.24**	1.00			
Perceived Competence	**0.21**	−0.14	**0.19**	**0.23**	**0.25**	**0.20**	1.00		
Depression	**−0.50**	**0.66**	**−0.60**	**−0.57**	**−0.49**	**−0.23**	**−0.21**	1.00	
Global Cognition	0.04	−0.09	0.09	0.05	0.06	0.11	0.05	−0.10	1.00

p-values < .05 are bolded

**Table 2:** Sample Demographic Characteristics, Functional Abilities, and Psychological Characteristics.

	Overall Sample (n = 277)

**Mean Age (SD)**	72.6 (5.1)
**Mean Years of Education (SD)**	15.9 (2.3)
**Female Gender (%)**	177 (63.9)
**Race/Ethnicity**	
**White; N (%)**	200 (72.2)
**Black; N (%)**	23 (8.3)
**Latino; N (%)**	34 (12.3)
**Other; N (%)**	20 (7.2)
**Positive Affect**	3.1 (0.7)
**Negative Affect**	1.7 (0.5)
**Life Satisfaction**	25.1 (5.8)
**Purpose in Life**	15.0 (2.1)
**Resiliency**	3.6 (0.7)
**Self-Management of Health**	3.5 (0.5)
**Perceived Competence**	23.5 (4.1)
**Global Cognition**	0.007 (1.0)
**Depression**	8.9 (7.8)
**Functional Abilities**	0.1 (0.2)

**Table 3:** Individual Regression results for Functional Abilities and Psychological Characteristics controlling for age, gender, and education.

Variable	Estimate (SE)	95 % CI	Standardized beta	p-value

**Negative Affect** ^a^	0.37 (0.08)	0.22 to 0.52	0.28	< 0.001 * **
**Positive Affect** ^a^	−0.31 (0.06)	−0.44 to −0.18	−0.28	< 0.001 * **
**Life Satisfaction** ^b^	−0.02 (0.008)	−0.03 to −0.004	−0.15	0.01 *
**Purpose in Life** ^b^	−0.06 (0.02)	−0.1 to −0.02	−0.16	0.007 * *
**Resiliency** ^b^	−0.21 (0.10)	−0.33 to −0.08	−0.19	0.002 * *
**Self-Management of Health** ^c^	−0.25 (0.10)	−0.44 to −0.06	−0.16	0.01 *
**Perceived Competence** ^d^	−0.01 (0.01)	−0.03 to 0.01	−0.06	0.34
**Global Cognition** ^b^	−0.12 (0.05)	−0.21 to −0.02	−0.16	0.02 *
**Depression** ^b^	0.03 (0.006)	0.02 to 0.04	0.28	< 0.001 * **

* p < .05

* * p < .01

* ** p < .001

a n = 275

b n = 274

c n = 268

d n = 273

Note: Estimates are unstandardized slopes (coefficients in the linear regression model), and the dependent variable is the log of the functional ability measure. Standardized betas are also presented.

**Table 4:** Final Linear Regression Model for Functional Abilities and Psychological Characteristics controlling for age, gender, education, global cognition, and depression.

Variable	Estimate (SE)	95 % CI	Standardized beta	p-value	VIF

**Age**	−0.004 (0.009)	−0.02 to 0.01	−0.02	0.68	1.13
**Education**	−0.004 (0.02)	−0.04 to 0.03	−0.01	0.84	1.11
**Male**	0.02 (0.09)	−0.16 to 0.20	0.01	0.85	1.10
**Negative Affect**	0.23 (0.11)	0.01 to 0.44	0.17	0.04 *	1.98
**Positive Affect**	−0.26 (0.08)	−0.42 to −0.09	−0.23	0.002 * *	1.63
**Life Satisfaction**	0.01 (0.01)	−0.006 to 0.03	0.10	0.18	1.78
**Purpose in Life**	0.002 (0.03)	−0.05 to 0.05	0.006	0.93	1.59
**Resiliency**	−0.01 (0.08)	−0.17 to 0.15	−0.01	0.90	1.71
**Self-Management of Health**	−0.10 (0.10)	−0.30 to 0.09	−0.07	0.29	1.14
**Global Cognition**	−0.09 (0.05)	−0.18 to 0.0001	−0.13	0.05^+^	1.23
**Depression**	0.009 (0.009)	−0.009 to 0.03	0.09	0.35	2.89

+ p < .10

* p < .05

* * p < .01; R^2^ = 15.6 %; Adjusted R^2^ = 12.0 %; n = 267

Note: Estimates are unstandardized slopes (coefficients in the linear regression model), and the dependent variable is the log of the functional ability measure. Standardized betas are also presented.

**Table 4b:** Final Linear Regression Model for Functional Abilities and Psychological Characteristics controlling for age, gender, education, and global cognition (depression dropped).

Variable	Estimate (SE)	95 % CI	Standardized beta	p-value	VIF

**Age**	−0.003 (0.009)	−0.02 to 0.01	−0.02	0.74	1.12
**Education**	−0.006 (0.02)	−0.04 to 0.03	−0.02	0.76	1.10
**Male**	0.02 (0.09)	−0.16 to 0.19	0.01	0.87	1.10
**Negative Affect**	0.28 (0.09)	0.10 to 0.46	0.21	0.003 * *	1.42
**Positive Affect**	−0.28 (0.08)	−0.43 to −0.12	−0.24	< 0.001 * **	1.52
**Life Satisfaction**	0.01 (0.01)	−0.008 to 0.03	0.08	0.27	1.59
**Purpose in Life**	−0.000 (0.02)	−0.05 to 0.05	−0.001	0.99	1.57
**Resiliency**	−0.02 (0.08)	−0.18 to 0.14	−0.02	0.78	1.67
**Self-Management of Health**	−0.10 (0.10)	−0.29 to 0.09	−0.06	0.30	1.13
**Global Cognition**	−0.09 (0.05)	−0.18 to 0.00	−0.13	0.05^+^	1.23

+ p < .10

* p < .05

* * p < .01

* ** p < .001; R^2^ = 15.4 %; Adjusted R^2^ = 12.0 %; n = 267

Note: Estimates are unstandardized slopes (coefficients in the linear regression model), and the dependent variable is the log of the functional ability measure. Standardized betas are also presented.
